# Microplastic Contamination in Salted Dried Beef

**DOI:** 10.1155/sci5/4994729

**Published:** 2026-06-03

**Authors:** Brenda Serrão de Freitas, Gabriel dos Anjos Guimarães, Gustavo Frigi Perotti, Beatriz Rocha de Moraes, Rômulo Augusto Ando, Gustavo Yomar Hattori, Bruno Sampaio Sant’Anna

**Affiliations:** ^1^ Institute of Exact Sciences and Technology, Federal University of Amazonas, Itacoatiara, Amazonas, Brazil, ufam.edu.br; ^2^ Institute of Geosciences, Federal University of Pará, Belém, Pará, Brazil, ufpa.br; ^3^ Department of Fundamental Chemistry, Institute of Chemistry, University of São Paulo, São Paulo, São Paulo, Brazil, usp.br

**Keywords:** environmental regulation, food, food safety, health risk, plastic pollution, sea salt

## Abstract

Plastic pollution is considered a major environmental challenge, and microplastics may pose risks to ecosystems and human health. This study investigated the presence of microplastics in salted dried beef sold in two cities in the Central Amazon, Brazil. Six brands, three sold in Itacoatiara and three sold in Parintins, were analyzed using density separation, organic matter digestion, stereomicroscopy, and Raman spectroscopy. All the samples showed contamination by microplastics, totaling 141 particles. In the brands from Itacoatiara, 55 particles were identified, with an average size of 1.85 ± 1.48 mm, while in the brands from Parintins, 86 particles were found, with an average size of 1.30 ± 1.17 mm. Elongated blue fibers were the most frequent in both locations. Chemical analysis identified four types of polymers: polyethylene terephthalate, acrylonitrile butadiene styrene, polystyrene, and polypropylene, which are mainly associated with urban sources. In this first study to demonstrate the contamination of salted dried beef by microplastics, the results reveal weaknesses in sanitary control processes and food safety, indicating potential risks to consumer health. This scenario reinforces the need to include microplastics as a parameter in sanitary surveillance programs and public food safety policies in the Central Amazon.

## 1. Introduction

Plastic pollution is recognized as one of the major global environmental threats due to its widespread presence in ecosystems and its potential risks to human and environmental health [1]. Since its introduction to the market in the 1950s, plastics have become essential in virtually all industrial sectors, driven by their physicochemical properties, low production cost, lightness, mechanical resistance, and high versatility [2]. This massive use has resulted in an exponential increase in global production, which rose from 1.5 million tons in 1950 to about 400 million tons in 2022 [3, 4]. Estimates indicate that if current production and disposal rates are maintained, approximately 730 million tons of plastic waste could accumulate in the environment by 2060 [5]. The low biodegradation rate of most plastic polymers causes these materials to persist for decades or even centuries, affecting various environmental compartments [6].

The most widely produced plastic polymer in the world is polyethylene (PE), and it accounts for about 26% of global plastic production. Other largely produced polymers include polypropylene (PP, 19%), polyvinyl chloride (PVC, 13%), polyurethane (PUR, 5%), polystyrene (PS, 5%), and PE terephthalate (PET, 2%) [7]. Once discarded, these materials are subject to degradation processes such as photodegradation, mechanical abrasion, and hydrolysis, which results in fragmentation into particles ranging from 0.1 μm to 5 mm, known as microplastics (MP) [8]. These fragments are widely distributed in aquatic and terrestrial environments and the atmosphere, representing a diffuse form of pollution with a high potential for bioaccumulation in food webs [9, 10].

Several studies have reported the presence of MP in different environmental matrices, such as surface waters [11], sediments [12, 13], soils [14], air [15], and plants [16], as well as in a wide variety of aquatic and terrestrial organisms [17–20]. The detection of MP in foods of plant and animal origin, such as vegetables [21], fish [22], shrimp [23, 24], bivalves [15, 25], honey [26], and milk [27], demonstrates their integration into the human food chain. The effects of these contaminants on living organisms manifest themselves at multiple biological levels, including biochemical, behavioral, and physiological alterations, potentially resulting in malformations and the development of diseases such as cancer [28]. In humans, the ingestion of foods contaminated with MP can trigger oxidative stress, as well as immune, respiratory, and cardiovascular disorders and even cancer [29, 30].

Among the foods that are widely contaminated by MP, commercial salt stands out [31, 32]. This product, essential to the human diet, is obtained from various sources, such as seawater, rock salt, and salt lakes, with sea salt being particularly prone to MP contamination due to its origin and its production process [33]. The evaporation of saltwater in open tanks, a common method in traditional salt production, facilitates the incorporation of MP present in the saltwater into the salt crystals [34]. Studies reveal that 90% of commercial salt samples analyzed in 35 countries contained MP [35], which heightens concerns about food safety, given the high average global salt consumption of 9–10 g/day, which exceeds the limit recommended by the World Health Organization (5 g/day) [36].

Salted and dried animal‐derived foods are widely consumed in various regions of the world due to their extended shelf life, ease of transport, and cultural significance, especially in areas with limited refrigeration [37]. These products can be contaminated with MP through multiple pathways, including the salts used in curing, the raw food itself, and during processing, handling, and packaging [38]. In the Amazon, for example, salted pirarucu (*Arapaima gigas* (Schinz, 1822)) holds significant cultural importance and is incorporated into various regional dishes [39]. During the salting process, large amounts of salt are applied directly to the food for periods that can exceed 10 days, increasing the risk of transferring contaminants present in the salt to the final product [40]. Studies indicate that MP contamination in salted shrimp sold in the Central Amazon is strongly associated with the use of contaminated salt during processing, representing a significant pathway for these pollutants to enter the food chain [24]. The presence of MP in widely consumed traditional foods highlights potential risks to food safety and public health, particularly in regions where sanitary inspection is limited and consumption of salted and dried products is frequent.

In this context, it is essential to systematically assess the occurrence of MP in salt‐preserved foods. To date, the presence of MP in salted dried beef has not been characterized. The aim of this study was to investigate the occurrence of MP in salted dried beef samples sold in two cities in the interior of the state of Amazonas, Brazil, compare contamination levels among different brands, and evaluate the potential public health risks associated with the consumption of these products. By addressing a traditional food of significant cultural and economic importance in the region, this study provides novel data on human exposure to MP in animal‐derived food products.

## 2. Materials and Methods

### 2.1. Sample Collection and Processing

Six brands of salted dried beef sold in the municipalities of Itacoatiara and Parintins, in the state of Amazonas, Brazil, were selected, with three brands from each location. For each brand, three 200 g samples were purchased, totaling 18 samples. The samples were transported to the laboratory in their original packaging, properly labeled with information about the brand, price, and place of origin, in order to ensure traceability during the analyses.

Each 200 g sample was subdivided into four 50‐g subsamples for analysis of MP. As a pretreatment, each subsample was placed in a 200 mL beaker containing filtered distilled water sufficient to completely cover the meat, in order to perform a preliminary wash. After 2 h, the meat was removed, and 50 mL of 30% (v/v) hydrogen peroxide (H_2_O_2_) solution was added to the beaker to promote the digestion of organic matter, following the methodology adapted [23, 24].

The samples with the oxidizing solution were incubated in an oven at 65°C for a period of 24–72 h, with periodic agitation to optimize the digestion of organic material. After this process, the resulting solution was filtered using microporous filter paper (porosity of 5 μm). The filters were then placed in Petri dishes, properly labeled, and left in a controlled environment for drying and subsequent microscopic analysis. To minimize the risk of cross‐contamination, all glassware used was thoroughly cleaned with distilled water and 70% (v/v) alcohol and subsequently covered with aluminum foil until the time of use. In addition, all solutions used were filtered through microporous filter paper (5 μm pore size) prior to use to prevent cross‐contamination. To monitor potential contamination during sample processing, 10 Petri dishes with clean filter paper were left exposed in the laboratory during the sample analyses. These were examined under a stereomicroscope, and no MP contamination was detected [23, 24].

### 2.2. Identification of MP

The filter paper with the samples was examined under a stereomicroscope (Leica EZ4) at magnifications ranging from 13× to 56× to check for the presence of MP. Particle sizes were measured (in mm) using the Motic Images Plus 2.0 mL analysis system, equipped with a camera (Moticam 2300 3.0) attached to the stereomicroscope. The MP particles were classified according to size, type, shape, and color. Within the type category, fragments, pellets, and filaments were considered. The smallest particle size characterized in this study was 0.1 mm. In the shape category, fragments were classified as round, irregular, subangular, or angular. Pellets were classified as disks, cylindrical, flat, spheroidal, or ovoid. Filaments were classified as elongated. For color, the classifications were crystalline, red, blue, black, orange, brown, yellow, transparent, white, opaque, and tan [41].

The identification of polymers present in the MP particles was carried out at the Spectroscopy Laboratory of the Department of Fundamental Chemistry at the Institute of Chemistry, University of São Paulo (IQ‐USP). For this stage, 16 samples that showed the highest abundance of MP in the preliminary analyses were selected and sent for spectroscopic characterization. The average particle size of the submitted MP was 1.84 ± 1.34 mm. The analyses were conducted using Raman spectroscopy. Raman spectra were obtained using a Renishaw inVia Micro‐Raman spectrometer, with an excitation wavelength of 785 nm and a 100x objective [22]. The obtained spectra were compared with reference databases compiled by the authors in order to determine the types of polymers present in the analyzed samples.

### 2.3. Statistical Analysis

The data regarding the number of MP particles by color, size, and shape, obtained for each salted dried beef brand from the cities of Itacoatiara and Parintins, were initially subjected to the Shapiro–Wilk test of normality. As the data did not show a normal distribution, nonparametric statistical tests were chosen. To compare the size and number of MP particles among different brands from each city, the Kruskal–Wallis test was used. When statistically significant differences were identified, the Dunn test was applied as a post hoc test for multiple comparisons between groups. For comparisons between the cities (Itacoatiara and Parintins), regardless of the brand, the Mann–Whitney test was employed to assess differences in the size and number of MP particles in the analyzed samples. In all the statistical analyses, a significance level of *p* <  0.05 was adopted.

## 3. Results

**FIGURE 1:**
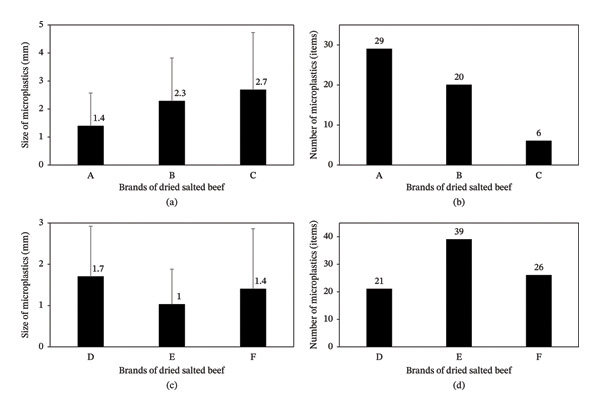
Distribution of microplastics in salted dried beef samples. (a) Particle size with mean and standard deviation in millimeters, found per brand in the city of Itacoatiara. (b) Number of particles found per brand in the city of Itacoatiara. (c) Particle size with mean and standard deviation in millimeters, found per brand in the city of Parintins. (d) Number of particles found per brand in the city of Parintins.

In the brands purchased in the city of Itacoatiara, a total of 55 MP particles were found, with an average length of 1.85 ± 1.48 mm. On average, the MP particles from Brand C were larger than those from the other brands purchased in Itacoatiara (Figure 1a). A significant difference was observed in the sizes of MP found among the brands from Itacoatiara (*H* = 7.3020; *P* = 0.0260). The highest number of MP per brand was recorded in Brand A, with 29 particles per 200 g of meat (Figure 1b). Analysis of the abundance of MP per sample did not reveal a significant difference in the quantities found (*H* = 4.7283; *P* = 0.0940).

In the brands purchased in the city of Parintins, a total of 86 MP particles were identified, with an average length of 1.30 ± 1.17 mm. On average, the particles from Brand D were larger compared to the other analyzed brands (Figure 1c); however, no significant differences were observed in the particle sizes (*H* = 5.0493; *P* = 0.0801). Brand E had the highest number of particles, with 39 per 200 g of meat, while the other brands showed lower contamination (Figure 1d). Analysis of abundance per sample also did not indicate a significant difference among the brands (*H* = 1.4237; *P* = 0.4907).

Considering all the samples from each city, regardless of the brand, a significant difference was found in the size of MP particles (*Z* (*U*) = 2.2719; *P* = 0.0231), but no significant difference was observed in the abundance of particles per sample.

**FIGURE 2:**
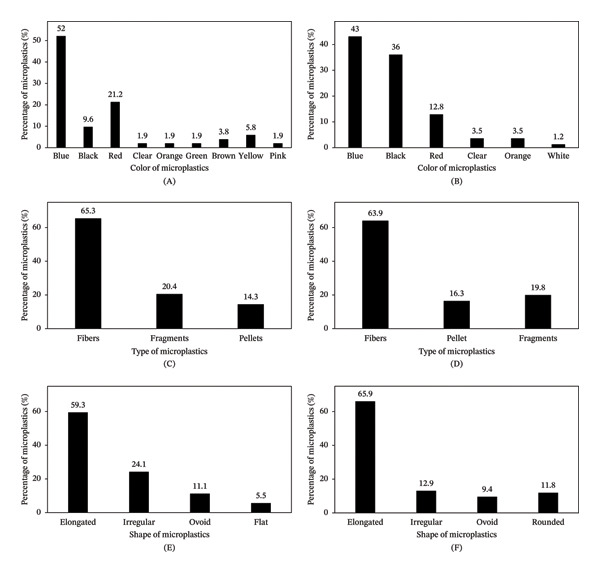
Morphology of the microplastics in salted dried beef from the cities of Itacoatiara and Parintins. Percentage distribution according to the color of the microplastics in salted dried beef samples from Itacoatiara (A) and Parintins (B). Percentage distribution according to type in samples from the respective cities: Itacoatiara (C) and Parintins (D). Percentage distribution according to the shape of microplastics in samples from Itacoatiara (E) and Parintins (F).

MP particles of various colors were identified, which included white, black, brown, orange, green, yellow, crystalline, and pink. The predominant color was blue, representing 52% in the brands from Itacoatiara (Figure 2A) and 43% in the brands from Parintins (Figure 2B). The most common type of particle found in all the brands was filaments (fibers), accounting for 65% in the brands from Itacoatiara (Figure 2C) and 64% in the brands from Parintins (Figure 2D). Fragments and pellets were also found (Figure 2C–D). Regarding shape, elongated particles were the most abundant, with 59% in the meats from Itacoatiara (Figure 2E) and 66% in those from Parintins (Figure 2F). In addition, irregular and ovoid‐shaped particles were identified (Figure 2E–F).

**FIGURE 3:**
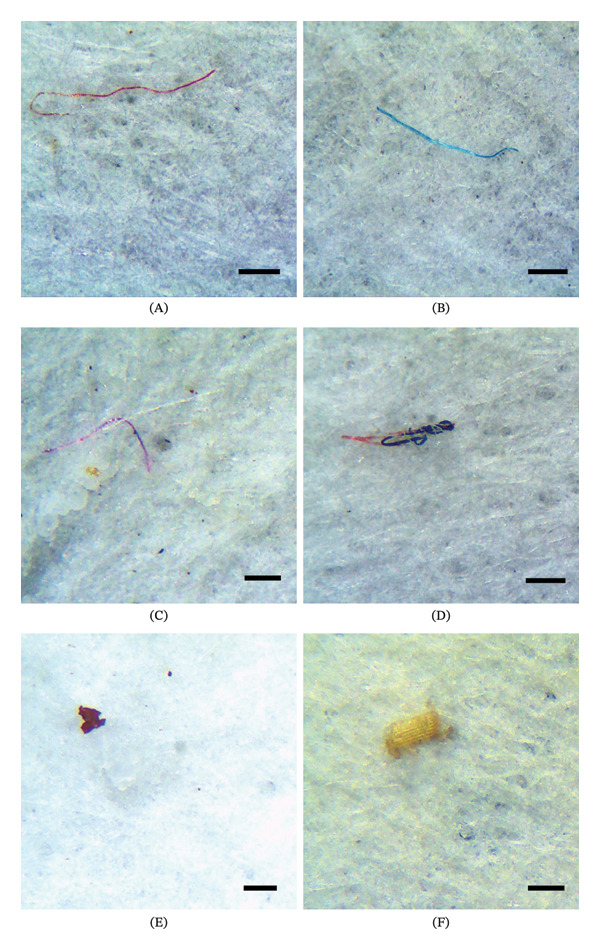
Most frequent microplastics found in salted dried beef samples collected in Itacoatiara. (A) Elongated red filament, (B) elongated blue filament, (C) elongated pink filament, (D) tangled filaments in blue and red, (E) irregular red fragment, and (F) irregular yellow fragment. Scale bar = 1 mm.

**FIGURE 4:**
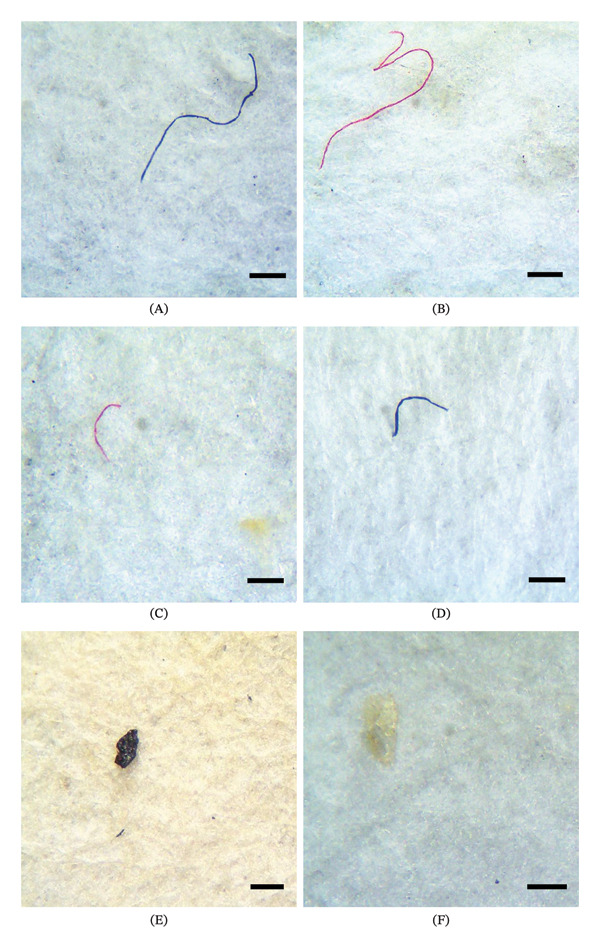
Most frequent microplastics found in salted dried beef samples collected in Parintins. (A) Elongated blue filament, (B) elongated red filament, (C) elongated red filament, (D) elongated blue filament, (E) irregular black fragment, and (F) irregular crystalline fragment. Scale bar = 1 mm.

The prices for salted dried beef in Itacoatiara ranged from US$ 5.60 to US$ 12.15 per kilogram. The lower priced brands showed a higher number of particles, as observed in Brand A. On the other hand, in Itacoatiara, Brand C, with a higher price, exhibited lower MP contamination. In Parintins, no price‐related pattern was identified, with prices varying slightly between US$ 8.41 and US$ 9.35 per kilogram, being similar across all brands, while MP contamination varied independently of price. Examples of MP with different sizes, shapes, types, and colors are shown in Figure 3 for the samples from Itacoatiara and in Figure 4 for the samples from Parintins.

**FIGURE 5:**
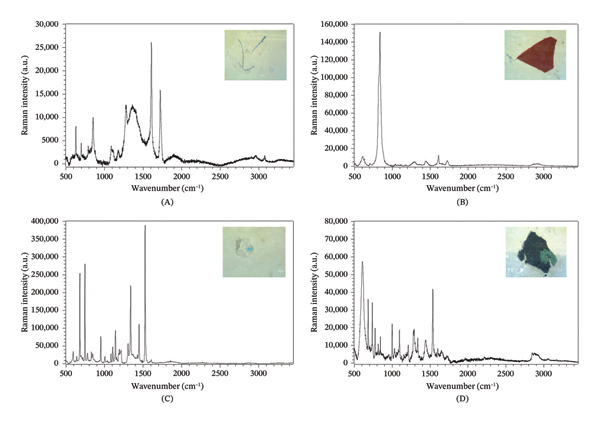
Representative examples of the various categories of microplastics identified using Raman spectroscopy. (A) Blue fiber (polyethylene terephthalate), (B) red fragment (polyethylene terephthalate), (C) blue fragment (acrylonitrile butadiene styrene), and (D) black fragment (mixture of polystyrene and polypropylene).

Based on the samples analyzed using Raman spectroscopy, the most common polymeric compounds identified were PET (34%), PE (22%), PP (11%), PS (11%), PUR (11%), and acrylonitrile butadiene styrene (ABS) (11%) of the total MPs analyzed. The blue fibers (Figure 5A) and red fragments (Figure 5B) were characterized as PET. The band at 854 cm^−1^ was attributed to out‐of‐plane bending of the C–H group and C–C bond stretching, while the band at 1089 cm^−1^ corresponds to the symmetric and asymmetric stretching of the C–O–C vibration, typical of the ester structure. The band at 1610/1613 cm^−1^ was associated with aromatic ring C=C stretching, confirming the presence of the aromatic group in PET. Finally, the bands at 1725/1730 cm^−1^ and 1280/1293 cm^−1^ were attributed to the stretching modes ν (C=O) (ester carbonyl) and ν (C (O)–O) (ester bond), respectively, corroborating the identification of the PET polymer structure [42, 43].

The blue fragment samples investigated using Raman spectroscopy were identified as ABS (Figure 5C). The band at 1008 cm^−1^ is attributed to the ring‐breathing vibration of the benzene ring in the styrene–acrylonitrile fraction of ABS. The band at 1610 cm^−1^ corresponds to the symmetric stretching mode of the C=C bonds in the benzene ring [44, 45]. However, it is important to note that the spectrum showed a large number of very intense signals at 680, 747, 952, 1144, 1342, 1450, and 1529 cm^−1^, which were attributed to the pigment Blue 15, a copper phthalocyanine [46], making it difficult to assign the polymer into which this pigment was dispersed.

The samples of black fragments analyzed by Raman spectroscopy were characterized as a mixture of PS and PP (Figure 5D). In the PS, a strong band at 1031 cm^−1^ was highlighted, attributed to the ring‐breathing mode of the aromatic carbon ring, a characteristic feature of this polymer. Other relevant bands included the skeletal ring stretching at 1602 cm^−1^ and the symmetric stretching modes of the aliphatic CH_2_ group at 2850 cm^−1^. Additionally, PS showed overlapping bands at 3059 cm^−1^, related to the stretching of the C–H bonds in the benzene ring [42, 47]. The PP, on the other hand, exhibited intense vibrations at 817 cm^−1^, associated with CH_2_ group rocking and C–C and C–CH_3_ stretching, and at 845 cm^−1^, related to CH_2_ and CH_3_ group rocking, as well as C–C and C–CH_3_ stretching. The band at 2879 cm^−1^ was attributed to symmetric stretching of the CH_3_ group [23, 42]. Similar to sample 5C, there was the presence of a large number of very intense bands, notably at 685, 741, 776, 818, 1214, 1283, 1339, 1390, 1446, and 1538 cm^−1^, which were attributed to the green phthalocyanine pigment (PG7) [48].

## 4. Discussion

It is widely recognized that fragmentation of plastics constitutes the main pathway for the dispersal of these residues in various environments, particularly in the form of micro‐ and nanoplastics [49]. In the present study, MP contamination was detected in all analyzed salted dried beef samples, highlighting the pervasive presence of these contaminants in traditional food products. The observed MP occurrence may result from multiple contamination pathways throughout the production and processing chain, including handling, transportation, and packaging, which are factors known to substantially increase MP levels in food [38]. Moreover, previous studies have indicated that sea salt represents a significant route for MP entry into the food chain [50], particularly in salted products. This issue is especially relevant in the Amazon region, where traditional foods such as dried shrimp [24] and the salted dried beef analyzed in this study are commonly consumed and generally subjected to salting with sea salt, a process that may facilitate MP contamination.

As observed in this study, where all analyzed salted meat brands were contaminated with MP, totaling 141 particles, previous research has also revealed concerning levels of contamination in widely consumed foods. For instance, Seth and Shriwastav [51] detected 626 MP particles across all evaluated salt brands, while Vidyasakar et al. [52] reported concentrations of 1075 particles. Similarly, Suteja et al. [53] found 520 particles in sea salt samples. MPs have also been detected in a wide range of meat products at levels exceeding 30,000 particles per kilogram [38]. The contamination may arise from multiple sources, including the salts used in processing, the meat itself, and the packaging materials. For example, meat stored in XPS trays may be contaminated with MP at levels ranging from 4.0 to 18.7 MP‐XPS/kg of packaged meat [54]. The contamination may arise from multiple sources, including the salts used in processing, the meat itself, and the packaging materials. For example, meat stored in extruded polystyrene trays may be contaminated with MP at levels ranging from 4.0 to 18.7 MP‐XPS/kg of packaged meat [54]. Considering the origins of the salts and the beef used in the six analyzed brands, these sources may collectively contribute to the MP load in the final products. These findings highlight the potential health risks associated with terrestrial animals intended for human consumption, especially given that beef and its derivatives are important sources of protein and essential nutrients [55].

Despite the significant amount of MP identified, the average size of the particles found in the present study was less than 2 mm. Lambert and Wagner [56] observed that the physical wear of plastics, even under exposure to visible light in situ, can promote the fragmentation of these materials into increasingly smaller particles, enhancing their spread in the environment. In analyses of salt, particles with primary sizes ranging from 0.1 to 2 mm were reported [53]. Similar sizes were also found in industrialized beverages, such as soft drinks, teas, and energy drinks, with particles ranging from 0.1 to 3 mm [57]. In packaged meats, Kedzierski et al. [54] recorded particles with dimensions of between 0.3 and 0.45 mm, values close to those observed in the salted dried beef samples analyzed in this study. The small size of these particles is inevitably one of the main factors favoring their wide dispersal, facilitating their penetration into containers, food, soils, and the environment as a whole [58].

The most frequent particles identified in the salted dried beef samples were blue, black, and red, present in all the analyzed brands from both cities. Studies such as those by Iñiguez et al. [59] and Yang et al. [60] also reported similar color patterns in MP found in commercial salt. In addition to these, other less predominant colors, such as yellow, orange, crystalline, pink, and green, were also observed in the samples of this study, which is consistent with the findings of Kim et al. [61], who identified a similar chromatic diversity in commercial salts. When analyzing MP in foods, Tokhun and Somparn [62] reported a predominance of blue, but also detected red, yellow, green, pink, violet, white, and transparent particles. The wide range of colors observed in various studies, including the present one, highlights the diversity of potential sources of contamination [63], reflecting the widespread use of colored plastics in daily life. The improper disposal of these materials significantly contributes to the presence of MP in natural environments, which ultimately reach the food chain [16].

Regarding morphology and typology, various terminologies are employed in the scientific literature [64]. In this study, three main morphological types were identified: filaments, pellets, and fragments. Filaments, also called fibers, were the predominant type in the analyzed salted dried beef samples, a finding consistent with several previous studies [60, 65–67]. In addition to filaments, pellets [60, 68] and fragments [60, 69] were also frequently found in all the evaluated salted meat brands.

The high concentration of fibers in the analyzed meat samples is primarily attributed to contamination from synthetic microfibers present in the commercial salts used during processing. Studies indicate that commercial salts contain high levels of microfibers [53], whose origin is directly associated with the release of MP during the washing of synthetic fabrics [13]. During these washing cycles, microfibers are released from textile fibers, entering wastewater systems, which are often inadequately treated or insufficiently treated, and consequently dispersed into aquatic environments such as rivers, lakes, and oceans [53, 70]. Napper and Thompson [71] demonstrated that a single washing cycle can release thousands of microfibers, predominantly composed of synthetic polymers such as polyester and nylon. Furthermore, the fibers present in salt may originate from the atmospheric deposition of MP transported by air during the crystallization process in an open environment [72].

In addition to fibers, MPs in the form of pellets were detected in 14%–16% of the meat samples. These pellets, classified as primary MPs, are industrially produced as raw material for various polymers and have a spherical or granular shape, optimized to facilitate transport and storage [72]. The entry of these MPs into the environment occurs through leaks during industrial processes, logistical failures, and improper waste disposal [73], which contributes to their high concentration in marine environments and, consequently, to the contamination of sea salt [60], an important vector of MPs into foods, including those analyzed in the present study.

MP fragments constituted a significant fraction of the MPs detected in the meat samples, characterized by their irregular morphologies and clear evidence of physical fragmentation of originally larger polymers [72]. These fragments are by‐products of the environmental degradation of macroplastics, such as plastic bags, bottles, rigid packaging, disposable utensils, and plastic toys, which undergo continuous fragmentation through abiotic and biotic mechanisms [53]. The global spread of plastic debris, especially in coastal and urbanized areas, intensifies the environmental load of these fragments [74]. Such accumulation contributes to substantial contamination of foods processed with sea salt [24, 75] along the meat supply chain [38], which is further reflected in the high abundance of MP fragments observed in the salted meat analyzed in this study.

The analysis of MP contamination in salted meat brands sold in Itacoatiara showed that lower cost products, such as Brand A, exhibited a higher concentration of MPs compared to higher‐priced products, such as Brand C. This result suggests that lower‐value products may be more susceptible to contamination, possibly due to the adoption of less rigorous quality control and hygiene practices during production processes. However, this relationship was not absolute, as higher‐priced brands from Parintins also showed high levels of MPs. This demonstrates that product price alone is not a reliable indicator of sanitary quality or compliance with good manufacturing practices.

The presence of PET MPs in the salted meat samples can be attributed to the fact that it is one of the most widely produced polymers worldwide [76]. Its use is widespread in the manufacture of plastic bottles, packaging, and synthetic fabrics, which promotes its broad environmental dispersion [77]. Similarly, ABS is a polymer widely used in industry for the production of electronic equipment, such as printers, vacuum cleaners, household appliances, musical instruments, and toys [78]. PS, in turn, is frequently employed in the manufacture of disposable products, such as cups, trays, food packaging, and thermal insulation materials, commonly found in the form of fragments or foam particles. PP is used in the production of bottle caps, straws, packaging, and various household utensils, and is one of the most recurrent polymers in environmental pollution due to its high resistance, durability, and widespread use [79].

The degradation and fragmentation of these materials, driven by physicochemical processes such as abrasion, ultraviolet radiation, and weathering, combined with improper disposal and transport by water currents [80, 81], promote their wide dispersal in aquatic and marine ecosystems. Recent studies show that these polymers are present not only in the water column and sediments but also in products intended for human consumption, such as commercial salts [53, 78, 82, 83]. This context reinforces that the presence of these MPs in the samples analyzed in the present study is a direct result of this environmental contamination cycle, which is reflected in food webs and, consequently, in the products consumed by the population, especially in the Amazon region.

In this context, a concerning weakness in sanitary and environmental control systems along the food production chain in the Amazon is evident [21, 24]. The presence of MPs in salted meat, regardless of the price range, raises serious concerns about compliance with food safety regulations, public health surveillance, and control of emerging contaminants [84, 85]. MP contamination can occur at different stages of the supply chain, including production, slaughter, transport, storage, and distribution. Implementing good practices and minimizing the use of plastic equipment and packaging are essential to reduce the entry of MP into beef products [55]. This also highlights limitations in public policies for food quality monitoring and environmental regulations, which should ensure not only food safety but also the protection of the health of the public against risks associated with contaminants such as MPs.

The direct impacts of human consumption of MPs, whether through salted dried beef, beverages, or other foods, are still poorly understood due to the complexity and novelty of the topic. However, studies in model organisms, including algae, zooplankton, fish, and mammals such as rats, show that exposure to MPs can trigger adverse responses, such as inflammatory disorders, immune dysfunctions, and metabolic alterations [86]. These findings indicate a potential risk to human health and serve as an early warning. In this context, the continuous ingestion of salted dried beef contaminated with MPs represents a potential pathway for chronic exposure for the population, raising concerns about long‐term toxic effects. In addition to the physical risk associated with the presence of the particles, the potential toxicity arising from chemical additives and organic contaminants adsorbed or incorporated into the MPs is noteworthy, as these may exacerbate impacts on human health [87, 88].

This study has some limitations that should be considered when interpreting the results. Additional controls, such as analytical‐grade salt or fresh unsalted beef, were not included and could have helped to more clearly distinguish potential sources of the detected MPs. Furthermore, the study design did not allow the specific origin of the particles to be traced along the production chain. Considering the relatively large size of some particles observed, it is plausible that part of the contamination may have occurred during processing, handling, packaging, or storage of the product.

Future studies should systematically investigate the sources and pathways of MP contamination throughout the entire salted beef production chain, from raw materials to final packaged products. This includes separate analyses of raw materials, such as different types of salt, analytical‐grade salt, and fresh beef, which could reveal primary sources of contamination. Detailed evaluations of processing stages, equipment, contact surfaces, and storage conditions are also crucial to identify critical control points where MPs may be introduced. In addition, research should assess the potential health risks to consumers, particularly focusing on chemical contaminants adsorbed onto or incorporated within MPs, which may amplify toxicological impacts. By integrating these analyses, future studies will provide essential information to develop evidence‐based mitigation strategies and best practices in the food industry, strengthen sanitary surveillance, and improve regulatory frameworks aimed at reducing MP contamination. Such efforts are vital not only to protect consumer health but also to ensure the safety, quality, and integrity of meat products in a highly complex production and distribution system.

## 5. Conclusion

This study highlights the presence of MPs in salted dried beef sold in the Central Amazon, emphasizing a significant public health and environmental concern. All the brands of salted meat were contaminated with MPs, with brands from Parintins showing higher levels of contamination compared to those from Itacoatiara. The average size of the MP particles found in the present study was less than 2 mm. The main colors of MPs identified were white, black, brown, orange, green, yellow, crystalline, and pink, with blue being the predominant color in both Itacoatiara and Parintins. The most common type of particle found across all brands was filaments. Regarding shape, elongated particles were the most abundant in meats from both Itacoatiara and Parintins. Four polymers were found in the samples: PET, ABS, PS, and PP. The results reveal concerning shortcomings in public health control systems and in handling and storage practices along the production chain, reflecting the fragility of food safety regulations regarding MP contamination. This contamination represents a potential risk to human health, as these materials can act as vectors for toxic substances and chemical contaminants. In this context, the study reinforces the urgent need to include MPs as a parameter in food safety surveillance and quality control programs, especially in the Amazon region, where there is increasing reliance on foods that are processed and stored for long periods. Additionally, it highlights the importance of strengthening public policies on solid waste management and environmental regulation to mitigate plastic emissions into the environment and reduce risks associated with food safety in the Central Amazon.

## Author Contributions

The first draft of the manuscript was written by Brenda S. Freitas, and this author also performed analyses of the separation of meat from microplastic particles. Gabriel A. Guimarães assisted in laboratory analysis to separate meat and microplastic particles and helped write the work. Gustavo F. Perotti helped with the interpretation of chemical analyses and writing the manuscript. Beatriz R. Moraes and Rômulo A. Ando were responsible for the analyses and interpretations in Raman spectroscopy. Gustavo Y. Hattori helped with general interpretations and writing of the manuscript. Bruno S. Sant’Anna designed and coordinated all research activities and contributed to writing the manuscript. All authors contributed to writing the final version of the manuscript.

## Funding

This study was financed by the Coordenação de Aperfeiçoamento de Pessoal de Nível Superior—Brasil (CAPES) and by the Fundo Brasileiro para a Biodiversidade (FUNBIO)—FUNBIO Scholarships Program—Conserving the Future, through scholarships for the author Gabriel dos Anjos Guimarães. Gustavo Yomar Hattori acknowledges the Fundação de Amparo à Pesquisa do Amazonas (FAPEAM) for funding through the Universal Amazonas Program (Grant No. 062.01258/2018) and PAINTER+ (Grant No. 062.00875/202001.02.016301.03900/2022‐66). Rômulo Augusto Ando and Beatriz Rocha de Moraes acknowledge the Fundação de Amparo à Pesquisa do Estado de São Paulo (Grant Nos. 2016/21070–5 and 2020/09250–3). The Conselho Nacional de Desenvolvimento Científico e Tecnológico provided a grant to Bruno Sampaio Sant’Anna (CNPq #409910/2016–3). Gustavo Frigi Perotti acknowledges FAPEAM for funding through PAINTER (Grant No. 062.00875/2020) and CT&I—Áreas Prioritárias (Grant No. 01.02.016301.03332/2021–12).

## Ethics Statement

The study was carried out in accordance with all applicable institutional, national, and international laws.

## Conflicts of Interest

The authors declare no conflicts of interest.

## Data Availability

The data that support the findings of this study are available from the corresponding author upon reasonable request.

## References

[1] United Nations Environment Programme (UNEP) , Nations Sign up to End Global Scourge of Plastic Pollution, UN News. (2022) https://news.un.org/en/story/2022/03/1113142.

[2] Akbulut S. , Akman P. K. , Tornuk F. , and Yetim H. , Microplastic Release From Single-Use Plastic Beverage Cups, Foods. (2024) 13, no. 10, 10.3390/foods13101564. PMC11121293 38790864

[3] Plastics Europe , The Compelling Facts About Plastics 2009, 2009, https://plasticseurope.org/knowledge-hub/plastics-the-facts-2009/.

[4] Plastics Europe , Plastics – The Fast Facts 2023, 2023, https://plasticseurope.org/knowledge-hub/plastics-the-fast-facts-2023/.

[5] Mai L. , Sun X. F. , Xia L. L. , Bao L. J. , Liu L. Y. , and Zeng E. Y. , Global Riverine Plastic Outflows, Environmental Science & Technology. (2020) 54, no. 16, 10049–10056, 10.1021/acs.est.0c02273. 32700904

[6] Ahmed N. , Utilizing Plastic Waste in the Building and Construction Industry: A Pathway Towards the Circular Economy, Construction and Building Materials. (2023) 383, 10.1016/j.conbuildmat.2023.131311.

[7] Houssini K. , Jinhui L. , and Quanyin T. , Complexities of the Global Plastics Supply Chain Revealed in a Trade-Linked Material Flow Analysis, Communications Earth & Environment. (2025) 6, 10.1038/s43247-025-02169-5.

[8] Jiang B. , Kauffman A. E. , Li L. et al., Health Impacts of Environmental Contamination of Micro- and Nanoplastics: A Review, Environmental Health and Preventive Medicine. (2020) 25, no. 1, 10.1186/s12199-020-00870-9. PMC7362455 32664857

[9] Jeong E. , Lee J. Y. , and Redwan M. , Animal Exposure to Microplastics and Health Effects: A Review, Emerging Contaminants. (2024) 10, no. 4, 10.1016/j.emcon.2024.100369.

[10] Gao C. , Xu B. , Li Z. et al., From Plankton to Fish: The Multifaceted Threat of Microplastics in Freshwater Environments, Aquatic Toxicology. (2025) 279, 10.1016/j.aquatox.2025.107242. 39799759

[11] Rico A. , Redondo-Hasselerharm P. E. , Vighi M. et al., Large-Scale Monitoring and Risk Assessment of Microplastics in the Amazon River, Water Research. (2023) 232, 10.1016/j.waters.2023.119707. 36773351

[12] Gerolin C. R. , Pupim F. N. , Sawakuchi A. O. , Grohmann C. H. , Labuto G. , and Semensatto D. , Microplastics in Sediments From Amazon Rivers, Brazil, Science of the Total Environment. (2020) 749, 10.1016/j.scitotenv.2020.141604. 32829281

[13] Oliveira L. G. , Hattori G. Y. , and Sant’Anna B. S. , Microplastic Contamination in Bathing Areas in the Central Amazon, Itacoatiara, Brazil, Environmental Science and Pollution Research. (2023) 30, no. 55, 117748–117758, 10.1007/s11356-023-30509-5. 37875761

[14] Long B. , Li F. , Wang K. , Huang Y. , Yang Y. , and Xie D. , Impact of Plastic Film Mulching on Microplastic in Farmland Soils in Guangdong Province, China, Heliyon. (2023) 9, no. 6, 10.1016/j.heliyon.2023.e16587. PMC10245015 37292288

[15] Rodrigues J. M. S. , Pantoja J. C. D. , Oliveira A. E. P. et al., First Evidence of Microplastics in Commercial Mussels From Amazonian Estuaries, Regional Studies in Marine Science. (2024) 70, 10.1016/j.rsma.2024.103379.

[16] Guimarães G. A. , Pereira S. A. , Moraes B. R. et al., The Retention of Plastic Particles by Macrophytes in the Amazon River, Brazil, Environmental Science and Pollution Research. (2024) 31, no. 30, 42750–42765, 10.1007/s11356-024-33961-z. 38877194

[17] Pegado T. S. S. , Schmid K. , Winemiller K. O. et al., First Evidence of Microplastic Ingestion by Fishes From the Amazon River Estuary, Marine Pollution Bulletin. (2018) 133, 814–821, 10.1016/j.marpolbul.2018.06.035. 30041381

[18] Correia L. L. , Ribeiro-Brasil D. R. G. , Garcia M. G. , Silva D. M. , Alencastre-Santos A. B. , and Vieira T. B. , The First Record of Ingestion and Inhalation of Micro- and Mesoplastics by Neotropical Bats From the Brazilian Amazon, Acta Chiropterologica. (2023) 25, no. 2, 371–383, 10.3161/15081109ACC2023.25.2.015.

[19] Firmino V. C. , Martins R. T. , Brasil L. S. et al., Do Microplastics and Climate Change Negatively Affect Shredder Invertebrates From an Amazon Stream? An Ecosystem Functioning Perspective, Environmental Pollution. (2023) 321, no. 2023, 10.1016/j.envpol.2023.121184. 36736567

[20] Souza-Ferreira M. L. C. , Reis A. J. O. , Ferreira E. B. L. , Dipold J. , Freitas A. Z. , Wetter N. U. , Oliveira-Bahia V. R. L. , and Vieira T. B. , First Record of Microplastic Contamination in Adult Endemic Amazonian Anuran Species, Scientific Reports. (2025) 15, no. 1, 10.1038/s41598-025-86434-9. PMC11743127 39827291

[21] Staffen H. C. S. , Guimarães G. A. , Hattori G. Y. , and Sant’Anna B. S. , Microplastics in Plant-Based Foods in the City of Itacoatiara (AM), Brazil, Brazilian Journal of Environmental Sciences. (2025) 60, 10.5327/Z2176-94782244.

[22] Azevedo I. J. G. , Moraes B. R. , Ando R. A. et al., Microplastics in Catfish *Pterygoplichthys pardalis* (Castelnau 1855) and *Hoplosternum littorale* (Hancock, 1828) Marketed in Itacoatiara, Amazonas, Brazil, Environmental Biology of Fishes. (2024) 107, no. 1, 107–119, 10.1007/s10641-024-01517-2.

[23] Guimarães G. A. , Moraes B. R. , Ando R. A. , Sant’Anna B. S. , Perotti G. F. , and Hattori G. Y. , Microplastic Contamination in the Freshwater Shrimp *Macrobrachium amazonicum* in Itacoatiara, Amazonas, Brazil, Environmental Monitoring and Assessment. (2023) 195, no. 3, 10.1007/s10661-023-11019-w. 36856928

[24] Guimarães G. A. , Moraes B. R. D. , Ando R. A. , Perotti G. F. , Sant’Anna B. S. , and Hattori G. Y. , Is the Shrimp *Macrobrachium amazonicum* Sold in an Urban Center in the Central Brazilian Amazon Contaminated With Microplastics?, Acta Amazonica. (2024) 54, no. 4, 10.1590/1809-4392202402002.

[25] Pantoja J. C. D. , Oliveira A. E. P. , Ferreira M. A. P. , Costa L. P. , Nunes Z. M. P. , and Rocha R. M. , First Register of Microplastic Contamination in Oysters (*Crassostrea Gasar*) Farmed in Amazonian Estuaries, Marine Pollution Bulletin. (2024) 201, 10.1016/j.marpolbul.2024.116182. 38382321

[26] Basaran B. , Özçifçi Z. , Kanbur E. D. et al., Microplastics in Honey From Türkiye: Occurrence, Characteristic, Human Exposure, and Risk Assessment, Journal of Food Composition and Analysis. (2024) 135, 10.1016/j.jfca.2024.106646.

[27] Zhang Q. , Liu L. , Jiang Y. et al., Microplastics in Infant Milk Powder, Environmental Pollution. (2023) 323, 10.1016/j.envpol.2023.121225. 36758924

[28] Barboza L. G. A. , Vieira L. R. , Branco V. et al., Microplastics Cause Neurotoxicity, Oxidative Damage and Energy-Related Changes and Interact With the Bioaccumulation of Mercury in the European Seabass, *Dicentrarchus Labrax* (Linnaeus, 1758), Aquatic Toxicology. (2018) 195, 49–57, 10.1016/j.aquatox.2017.12.008. 29287173

[29] Prata J. C. , da Costa J. P. , Lopes I. , Duarte A. C. , and Rocha-Santos T. , Environmental Exposure to Microplastics: An Overview on Possible Human Health Effects, Science of the Total Environment. (2020) 702, 10.1016/j.scitotenv.2019.134455. 31733547

[30] Segovia-Mendoza M. , Nava-Castro K. E. , Palacios-Arreola M. I. , Garay-Canales C. , and Morales-Montor J. , How Microplastic Components Influence the Immune System and Impact on Children Health: Focus on Cancer, Birth Defects Research. (2020) 112, no. 18, 1341–1361, 10.1002/bdr2.1779. 32767490

[31] Makhdoumi P. , Pirsaheb M. , Amin A. A. , Kianpour S. , and Hossini H. , Microplastic Pollution in Table Salt and Sugar: Occurrence, Qualification and Quantification and Risk Assessment, Journal of Food Composition and Analysis. (2023) 119, 10.1016/j.jfca.2023.105261.

[32] Nakat Z. , Dgheim N. , Ballout J. , and Bou-Mitri C. , Occurrence and Exposure to Microplastics in Salt for Human Consumption, Present on the Lebanese Market, Food Control. (2023) 145, 10.1016/j.foodcont.2022.109414.

[33] Zhang Q. , Xu E. G. , Li J. , Chen Q. , Zeng E. Y. , and Shi H. , A Review of Microplastics in Table Salt, Drinking Water, and Air: Direct Human Exposure, Environmental Science & Technology. (2020) 54, no. 7, 3740–3751, 10.1021/acs.est.9b04535. 32119774

[34] Hueso-Kortekaas K. , Delgado-Mellado N. , Calzada-Funes J. , Sanchez-Mata C. , Castañeda C. , and Cledera-Castro M. D. M. , Microplastics in Inland Saline Lakes of the Central Ebro Basin, NE Spain, Water. (2025) 17, no. 7, 10.3390/w17070989.

[35] Peixoto D. , Pinheiro C. , Amorim J. , Oliva-Teles L. , Guilhermino L. , and Vieira M. N. , Microplastic Pollution in Commercial Salt for Human Consumption: A Review, Estuarine, Coastal and Shelf Science. (2019) 219, 161–168, 10.1016/j.ecss.2019.02.018.

[36] Lan V. T. H. , Quyen B. T. T. , Duy P. Q. , Hoang L. , and Minh H. V. , Trends in Salt Consumption and Reduction Practices in Vietnam During 2015–2021: Analyzing Urinary Sodium Levels Among 18–69 Aged Populations, International Journal of Public Health. (2025) 70, 10.3389/ijph.2025.1608065. PMC11975537 40201421

[37] Belton B. , Johnson D. S. , Thrift E. , Olsen J. , Hossain M. A. R. , and Thilsted S. H. , Dried Fish at the Intersection of Food Science, Economy, and Culture: A Global Survey, Fish and Fisheries. (2022) 23, no. 4, 941–962, 10.1111/faf.12664.

[38] Rahman S. , Sarker P. , Datta T. R. et al., From Farm to Fork: Microplastic Contamination in the Meat and Dairy Supply Chain, Current Research in Food Science. (2026) 12, 10.1016/j.crfs.2026.101334. PMC12891781 41684710

[39] Silva S. O. , Junior L. P. G. , Machado M. B. et al., 1H NMR Spectroscopy as a Tool to Probe Potential Biomarkers of the Drying-Salting Process: A Proof-of-Concept Study With the Amazon Fish Pirarucu, Food Chemistry. (2024) 448, 10.1016/j.foodchem.2024.139047. 38520988

[40] Sabadini E. , Hubinger M. D. , Sobral P. J. A. , and Carvalho B. C.Jr., Alterações Da Atividade de Água e da Cor da Carne No Processo de Elaboração da Carne Salgada Desidratada, Food Science and Technology. (2001) 21, no. 1, 1–7, 10.1590/S0101-20612001000100005.

[41] Hanke G. , François G. , Werner S. et al., Guidance on Monitoring of Marine Litter in European Seas, 2013, European Union Publications Office.

[42] Nava V. , Frezzotti M. L. , and Leoni B. , Raman Spectroscopy for the Analysis of Microplastics in Aquatic Systems, Applied Spectroscopy. (2021) 75, no. 11, 1341–1357, 10.1177/00037028211043119. 34541936

[43] Peñalver R. , Zapata F. , Arroyo-Manzanares N. , Lopez-García I. , and Viñas P. , Raman Spectroscopic Strategy for the Discrimination of Recycled Polyethylene Terephthalate in Water Bottles, Journal of Raman Spectroscopy. (2023) 54, no. 1, 107–112, 10.1002/jrs.6457.

[44] Reggio D. , Saviello D. , Lazzari M. , and Iacopino D. , Characterization of Contemporary and Historical Acrylonitrile Butadiene Styrene (ABS)-Based Objects: Pilot Study for Handheld Raman Analysis in Collections, Spectrochimica Acta Part A: Molecular and Biomolecular Spectroscopy. (2020) 242, 10.1016/j.saa.2020.118733. 32731147

[45] Oubella M. , Ben Jadi S. , El Fazdoune M. et al., Effect of Surface Pretreatment on the Polypyrrole Coating of Acrylonitrile Butadiene Styrene. Part A: Synthesis and characterization, Progress in Organic Coatings. (2024) 195, 10.1016/j.porgcoat.2024.108654.

[46] Bovill A. J. , McConnell A. A. , Nimmo J. A. , and Smith W. E. , Resonance Raman Spectra of Alpha-Copper Phthalocyanine, Journal of Physical Chemistry. (1986) 90, no. 4, 569–575, 10.1021/j100276a017.

[47] Mayorga C. , Athalye S. M. , Boodaghidizaji M. et al., Limit of Detection of Raman Spectroscopy Using Polystyrene Particles from 25 to 1000 nm in Aqueous Suspensions, Analytical Chemistry. (2025) 97, no. 16, 8908–8914, 10.1021/acs.analchem.5c00182. 40228800 PMC12044590

[48] Faria D. L. D. and Puglieri T. S. , Diferenciando Reproduções e Pinturas Verdadeiras: Um Interessante Estudo de Caso, Química Nova. (2016) 39, no. 5, 542–547, 10.5935/0100-4042.20160056.

[49] Maghsodian Z. , Sanati A. M. , Tahmasebi S. , Shahriari M. H. , and Ramavandi B. , Study of Microplastics Pollution in Sediments and Organisms in Mangrove Forests: A Review, Environmental Research. (2022) 208, 10.1016/j.envres.2022.112725. 35063433

[50] Goswami S. , Adhikary S. , Bhattacharya S. et al., The Alarming Link Between Environmental Microplastics and Health Hazards With Special Emphasis on Cancer, Life Sciences. (2024) 355, 10.1016/j.lfs.2024.122937. 39103046

[51] Seth C. K. and Shriwastav A. , Contamination of Indian Sea Salts With Microplastics and a Potential Prevention Strategy, Environmental Science and Pollution Research. (2018) 25, no. 30, 30122–30131, 10.1007/s11356-018-3028-5. 30145764

[52] Vidyasakar A. , Krishnakumar S. , Kumar K. S. et al., Microplastic Contamination in Edible Sea Salt From the Largest Salt-Producing States of India, Marine Pollution Bulletin. (2021) 171, 10.1016/j.marpolbul.2021.112728. 34303058

[53] Suteja Y. , Dirgayusa I. G. N. P. , Purnama S. G. , and Purwiyanto A. I. S. , From Sea to Table: Assessing Microplastic Contamination in Local and Non-Local Salt in Bali, Indonesia, Chemosphere. (2025) 374, 10.1016/j.chemosphere.2025.144192. 39938321

[54] Kedzierski M. , Lechat B. , Sire O. , Le Maguer G. , Le Tilly V. , and Bruzaud S. , Microplastic Contamination of Packaged Meat: Occurrence and Associated Risks, Food Packaging and Shelf Life. (2020) 24, 10.1016/j.fpsl.2020.100489.

[55] Hantoro I. , Harumi M. , Ardanareswari K. , Soedarini B. , and Widianarko B. , The Invisible Threat: Assessing Microplastic Contamination in Beef and Its Implications for Food Safety, Environmental and Natural Resources Journal. (2026) 24, no. 2, 140–148, 10.32526/ennrj/24/20240348.

[56] Lambert S. and Wagner M. , Characterisation of Nanoplastics During the Degradation of Polystyrene, Chemosphere. (2016) 145, 265–268, 10.1016/j.chemosphere.2015.11.078. 26688263 PMC5250697

[57] Shruti V. C. , Pérez-Guevara F. , Elizalde-Martínez I. , and Kutralam-Muniasamy G. , First Study of Its Kind on the Microplastic Contamination of Soft Drinks, Cold Tea and Energy Drinks – Future Research and Environmental Considerations, Science of the Total Environment. (2020) 726, 10.1016/j.scitotenv.2020.138580. 32315857

[58] Sridhar A. , Kannan D. , Kapoor A. , and Prabhakar S. , Extraction and Detection Methods of Microplastics in Food and Marine Systems: A Critical Review, Chemosphere. (2022) 286, 10.1016/j.chemosphere.2021.131653. 34346338

[59] Iñiguez M. E. , Conesa J. A. , and Fullana A. , Microplastics in Spanish Table Salt, Scientific Reports. (2017) 7, no. 1, 10.1038/s41598-017-09128-x. PMC5561224 28819264

[60] Yang D. , Shi H. , Li L. , Li J. , Jabeen K. , and Kolandhasamy P. , Microplastic Pollution in Table Salts From China, Environmental Science & Technology. (2015) 49, no. 22, 13622–13627, 10.1021/acs.est.5b03163. 26486565

[61] Kim J. S. , Lee H. J. , Kim S. K. , and Kim H. J. , Global Pattern of Microplastics (Mps) in Commercial Food-Grade Salts: Sea Salt as an Indicator of Seawater MP Pollution, Environmental Science & Technology. (2018) 52, no. 21, 12819–12828, 10.1021/acs.est.8b04180. 30285421

[62] Tokhun N. and Somparn A. , Microplastic Contaminations in Buffet Food From Local Markets, Asian Health, Science and Technology Reports. (2020) 28, no. 4, 13–20, 10.14456/nujst.2020.32.

[63] Stolte A. , Forster S. , Gerdts G. , and Schubert H. , Microplastic Concentrations in Beach Sediments Along the German Baltic Coast, Marine Pollution Bulletin. (2015) 99, no. 1–2, 216–229, 10.1016/j.marpolbul.2015.07.022. 26198261

[64] Frias J. P. G. L. and Nash R. , Microplastics: Finding a Consensus on the Definition, Marine Pollution Bulletin. (2019) 138, 145–147, 10.1016/j.marpolbul.2018.11.022. 30660255

[65] Anderson P. J. , Warrack S. , Langen V. , Challis J. K. , Hanson M. L. , and Rennie M. D. , Microplastic Contamination in Lake Winnipeg, Canada, Environmental Pollution. (2017) 225, 223–231, 10.1016/j.envpol.2017.02.072. 28376390

[66] Kosuth M. , Mason S. A. , and Wattenberg E. V. , Anthropogenic Contamination of Tap Water, Beer, and Sea Salt, PLoS One. (2018) 13, no. 4, 10.1371/journal.pone.0194970. PMC5895013 29641556

[67] Corami F. , Rosso B. , Bravo B. , Gambaro A. , and Barbante C. , A Novel Method for Purification, Quantitative Analysis and Characterization of Microplastic Fibers Using Micro-FTIR, Chemosphere. (2020) 238, 10.1016/j.chemosphere.2019.124564. 31472348

[68] do Sul J. A. I. and Costa M. F. , The Present and Future of Microplastic Pollution in the Marine Environment, Environmental Pollution. (2014) 185, 352–364, 10.1016/j.envpol.2013.10.036. 24275078

[69] Bayo J. , Olmos S. , and López-Castellanos J. , Microplastics in an Urban Wastewater Treatment Plant: The Influence of Physicochemical Parameters and Environmental Factors, Chemosphere. (2020) 238, 10.1016/j.chemosphere.2019.124593. 31446275

[70] Queiroz L. G. , Pompêo M. , de Moraes B. R. , Ando R. , and Rani-Borges B. , Implications of Damming and Morphological Diversity of Microplastics in the Sediment From a Tropical Freshwater Reservoir, Journal of Environmental Chemical Engineering. (2024) 12, no. 2, 10.1016/j.jece.2024.112234.

[71] Napper I. E. and Thompson R. C. , Release of Synthetic Microplastic Fibres From Domestic Washing Machines: Effects of Fabric Type and Washing Conditions, Marine Pollution Bulletin. (2016) 112, no. 1-2, 39–45, 10.1016/j.marpolbul.2016.09.025. 27686821

[72] Lusher A. L. , Bråte I. L. N. , Munno K. , Hurley R. R. , and Welden N. A. , Is It or Isn’t It: The Importance of Visual Classification in Microplastic Characterization, Applied Spectroscopy. (2020) 74, no. 9, 1139–1153, 10.1177/0003702820930733. 32394728

[73] Lee A. , Mondon J. , Merenda A. , Dumée L. F. , and Callahan D. L. , Surface Adsorption of Metallic Species onto Microplastics With Long-Term Exposure to the Natural Marine Environment, Science of the Total Environment. (2021) 780, 10.1016/j.scitotenv.2021.146613. 34030302

[74] Pereira M. F. B. C. , Martinelli Filho J. E. , Lopez-Ibáñez S. , Gómez Salazar C. , and Beiras R. , In Situ Plastic Fragmentation Between Two Contrasting Ecosystems in the Atlantic, Marine Environmental Research. (2025) 208, 10.1016/j.marenvres.2025.107131. 40209624

[75] Venkatesh S. , Naidu B. C. , Palanisamy S. et al., Microplastic Accumulation Dynamics and Risk Assessment in Dried Fish Processed With Sea Salt at Different Salting Ratios, Journal of Hazardous Materials Advances. (2024) 14, 10.1016/j.hazadv.2024.100415.

[76] Soong Y. H. V. , Sobkowicz M. J. , and Xie D. , Recent Advances in Biological Recycling of Polyethylene Terephthalate (PET) Plastic Wastes, Bioengineering. (2022) 9, no. 3, 10.3390/bioengineering9030098. PMC8945055 35324787

[77] Sadeghi B. , Marfavi Y. , AliAkbari R. , Kowsari E. , Ajdari F. B. , and Ramakrishna S. , Recent Studies on Recycled PET Fibers: Production and Applications: A Review, Materials Circular Economy. (2021) 3, no. 1, 10.1007/s42824-020-00014-y.

[78] Ravikumar S. , Jeyameenakshi A. , Ali M. S. , and Ebenezer K. S. , Assessment of Microplastics in Edible Salts From Solar Saltpans and Commercial Salts, Total Environment Research Themes. (2023) 6, 10.1016/j.totert.2023.100032.

[79] Gangadharan G. , Bharti A. , and Mondal A. , Electrochemical Degradation Strategies for Polystyrene Microplastic: Current Trends and Future Prospects, Polymer Degradation and Stability. (2025) 238, 10.1016/j.polymdegradstab.2025.111351.

[80] Malli A. , Corella-Puertas E. , Hajjar C. , and Boulay A. M. , Transport Mechanisms and Fate of Microplastics in Estuarine Compartments: A Review, Marine Pollution Bulletin. (2022) 177, 10.1016/j.marpolbul.2022.113553. 35303633

[81] Belioka M. P. and Achilias D. S. , The Effect of Weathering Conditions in Combination With Natural Phenomena/Disasters on Microplastics’ Transport From Aquatic Environments to Agricultural Soils, Microplastics. (2024) 3, no. 3, 518–538, 10.3390/microplastics3030033.

[82] Kuttykattil A. , Raju S. , Vanka K. S. et al., Consuming Microplastics? Investigation of Commercial Salts as a Source of Microplastics (Mps) in Diet, Environmental Science and Pollution Research. (2023) 30, no. 1, 930–942, 10.1007/s11356-022-22101-0. 35907067 PMC9813175

[83] Maharjan K. K. and Dhungel R. P. , First-Ever Study Uncovers Microplastic Contamination in Nepalese Table Salt, Heliyon. (2024) 10, no. 14, 10.1016/j.heliyon.2024.e34621. PMC11284381 39082014

[84] Silva G. C. , Galleguillos Madrid F. M. , Hernández D. et al., Microplastics and Their Effect in Horticultural Crops: Food Safety and Plant Stress, Agronomy. (2021) 11, no. 8, 10.3390/agronomy11081528.

[85] Dini I. , Mancusi A. , and Seccia S. , From Harm to Hope: Tackling Microplastics’ Perils With Recycling Innovation, Molecules. (2025) 30, no. 12, 10.3390/molecules30122535. PMC12195888 40572499

[86] Hwang J. , Choi D. , Han S. , Choi J. , and Hong J. , An Assessment of the Toxicity of Polypropylene Microplastics in Human Derived Cells, Science of the Total Environment. (2019) 684, 657–669, 10.1016/j.scitotenv.2019.05.071. 31158627

[87] Gateuille D. and Naffrechoux E. , Transport of Persistent Organic Pollutants: Another Effect of Microplastic Pollution?, Wiley Interdisciplinary Reviews: Water. (2022) 9, no. 5, 10.1002/wat2.1600.

[88] Gerolin C. R. , Zornio B. , Pataro L. F. , Labuto G. , and Semensatto D. , Microplastic Pollution Responses to Spatial and Seasonal Variations and Water Level Management in a Polymictic Tropical Reservoir (São Paulo, Brazil), Environmental Science and Pollution Research. (2024) 31, no. 29, 42388–42405, 10.1007/s11356-024-33960-0. 38874755

